# Pitfalls of DNA Quantification Using DNA-Binding Fluorescent Dyes and Suggested Solutions

**DOI:** 10.1371/journal.pone.0150528

**Published:** 2016-03-03

**Authors:** Yuki Nakayama, Hiromi Yamaguchi, Naoki Einaga, Mariko Esumi

**Affiliations:** Department of Pathology, Nihon University School of Medicine, Itabashi-ku, Tokyo, Japan; Osaka University, JAPAN

## Abstract

The Qubit fluorometer is a DNA quantification device based on the fluorescence intensity of fluorescent dye binding to double-stranded DNA (dsDNA). Qubit is generally considered useful for checking DNA quality before next-generation sequencing because it measures intact dsDNA. To examine the most accurate and suitable methods for quantifying DNA for quality assessment, we compared three quantification methods: NanoDrop, which measures UV absorbance; Qubit; and quantitative PCR (qPCR), which measures the abundance of a target gene. For the comparison, we used three types of DNA: 1) DNA extracted from fresh frozen liver tissues (Frozen-DNA); 2) DNA extracted from formalin-fixed, paraffin-embedded liver tissues comparable to those used for Frozen-DNA (FFPE-DNA); and 3) DNA extracted from the remaining fractions after RNA extraction with Trizol reagent (Trizol-DNA). These DNAs were serially diluted with distilled water and measured using three quantification methods. For Frozen-DNA, the Qubit values were not proportional to the dilution ratio, in contrast with the NanoDrop and qPCR values. This non-proportional decrease in Qubit values was dependent on a lower salt concentration, and over 1 mM NaCl in the DNA solution was required for the Qubit measurement. For FFPE-DNA, the Qubit values were proportional to the dilution ratio and were lower than the NanoDrop values. However, electrophoresis revealed that qPCR reflected the degree of DNA fragmentation more accurately than Qubit. Thus, qPCR is superior to Qubit for checking the quality of FFPE-DNA. For Trizol-DNA, the Qubit values were proportional to the dilution ratio and were consistently lower than the NanoDrop values, similar to FFPE-DNA. However, the qPCR values were higher than the NanoDrop values. Electrophoresis with SYBR Green I and single-stranded DNA (ssDNA) quantification demonstrated that Trizol-DNA consisted mostly of non-fragmented ssDNA. Therefore, Qubit is not always the most accurate method for quantifying DNA available for PCR.

## Introduction

Recently, various simple quantification methods have been developed to determine DNA concentrations in trace amounts of samples. These techniques have been useful in medicine, including in molecular diagnosis and prognosis, e.g., the detection of cell-free fetal DNA in maternal circulation and circulating tumor cells [1–5]. Recently, clinical samples have also been subjected to novel technologies, such as next-generation sequencing (NGS) in translational research. It has become important to more accurately evaluate the quality and quantity of DNA. The following three methods are used to quantify DNA: 1) UV absorbance measurement at 260 nm (UV spectroscopy); 2) fluorescence measurement of a fluorescent dye, such as PicoGreen, specifically bound to double-stranded DNA (dsDNA) (fluorescence spectroscopy) [6]; and 3) relative quantification of a particular DNA sequence based on real-time PCR (quantitative PCR; qPCR) [7]. The UV spectroscopy method measures the maximal absorbance of nucleic acids; thus, it does not distinguish between dsDNA, single-stranded DNA (ssDNA), RNA and nucleotides. In contrast, the fluorescence spectroscopy method determines the amount of intact dsDNA, and the quantitative value yielded decreases with the level of fragmentation and denaturation of DNA [8–11]. Therefore, fluorescence spectroscopy using PicoGreen is more useful than UV spectroscopy for evaluating the template activity of DNA for PCR. In this regard, qPCR accurately quantifies the amount of the target sequence and is the ideal method for checking the quantity of DNA used for NGS [12]. However, qPCR takes much more time and is more expensive than fluorometry or UV spectroscopy. Simbolo M et al. have suggested that the ideal workflow for quantifying DNA, especially DNA extracted from histopathological tissues suitable for NGS, is first to assess the presence of contaminants in the sample with a UV spectrometer (NanoDrop) and subsequently to use a fluorescence spectrometer (Qubit) to quantify dsDNA [13]. However, it is unknown whether Qubit can be completely replaced by qPCR for determination of DNA concentrations.

In the present study, we quantified the following three types of DNA using the three quantification methods NanoDrop, Qubit and qPCR: DNA extracted from fresh frozen tissues, formalin-fixed paraffin-embedded (FFPE) DNA and DNA extracted from the remaining fraction after RNA extraction with Trizol reagent (Life Technologies). We found inconsistencies in DNA quantification between Qubit and qPCR and proposed the optimum combinations of the aforementioned DNA quantification methods.

## Materials and Methods

### Samples

Fresh frozen non-tumorous liver tissues were obtained from six patients with liver metastasis of colorectal carcinoma by surgical resection and from five Long Evans Cinnamon rats (Frozen-H1 to H6 and R1 to R5). The freshly frozen livers were stored at -80°C until use. FFPE samples were prepared from the same human liver tissues as described above (FFPE-H1 to H3) with fixation in 10% buffered formalin for 2 to 4 days. Human non-tumorous liver tissues were also obtained from the resection of seven cases with HCV-positive hepatocellular carcinoma (Trizol-h1 to h7), and RNA was extracted using Trizol reagent (Life Technologies, Waltham, MA USA). The fractions remaining after RNA extraction were stored (Trizol-h1 to h7) at -80°C until use. Our study protocol was approved by the Ethics Committee of the Nihon University School of Medicine in accordance with the 1975 Declaration of Helsinki, and written informed consent was obtained from each patient. Animal experiments were approved by the Nihon University Animal Care and Use Committee in accordance with the institutional animal care guidelines of Nihon University.

### DNA extraction

DNA was extracted from frozen liver tissues by the standard protocol using proteinase K and phenol, as described previously [14]. DNA was precipitated with isopropanol, and the DNA pellet was rinsed with 70% ethanol.

FFPE tissues (FFPE-H1 to H3) were sliced into 10-μm-thick sections. DNA was extracted from two sections of each sample using a RecoverAll Total Nucleic Acid Isolation Kit for FFPE (Life Technologies) according to the manufacturer’s protocol with some modifications; briefly, after deparaffinization and protease digestion, the samples were heated to 95°C for 30 minutes. DNA was finally eluted with 60 μl distilled water at 95°C twice. Then, DNA was precipitated with 0.3 M sodium acetate and 70% ethanol. The DNA pellet was rinsed with 70% ethanol.

The remaining fractions after RNA extraction with Trizol reagent (Trizol-h1 to h7) were subjected to DNA extraction according to the manufacturer’s protocol, with some modifications. Briefly, after the DNA was precipitated with 300 μl of 100% ethanol per 1 ml Trizol reagent used for the initial homogenization, the DNA pellet was washed with a mixture of 0.1 M sodium citrate/10% ethanol by vortex mixing three times at 10-minute intervals; the pellet was then rinsed twice with 75% ethanol. All DNA pellets were dissolved in distilled water.

### Quantification of DNA by three methods

The concentrations of all DNA solutions were determined using a NanoDrop-2000 (Thermo Fisher Scientific, Wilmington, DE, USA) and a Qubit 3.0 fluorometer (Life Technologies). S1 Table shows the DNA concentrations and OD_260_/OD_280_ and OD_260_/OD_230_ ratios of all original solutions, as determined by NanoDrop. A Qubit dsDNA BR (broad range, 2 to 1000 ng) Assay Kit and Qubit dsDNA HS (high sensitivity, 0.2 to 100 ng) Assay Kit were used with a Qubit 3.0 fluorometer according to the manufacturer’s protocols; a sample volume of 1 μl was added to 199 μl of a Qubit working solution.

The effective quantity of DNA for PCR was measured by qPCR targeting the genome sequences of human (157 bp) and rat (175 bp) *glyceraldehyde-3-phosphate dehydrogenase*; TaqMan Gene Expression Assays Hs02786624_g1 and Rno1775763_g1 (Life Technologies) were used. PCR was performed with a 10 μl reaction mixture containing TaqMan Fast Advanced Master Mix (Life Technologies) or Premix Ex Taq™ (Probe qPCR) (Takara Bio, Shiga, Japan), with an initial denaturation step at 95°C for 10 seconds, followed by 45 cycles at 95°C for 1 second and 60°C for 20 seconds. The quantitative values determined by qPCR were obtained as follows: the relative quantification ΔCT method was performed using NanoDrop quantification of Frozen-H1 and Frozen-R1 as standards for human and rat DNA quantification, respectively, because the CT values of all Frozen-DNAs were constant. The ratio of the sample quantity to the standard was calculated as 2^-ΔCT^, and the sample quantity was determined to be the formula of the standard NanoDrop quantity x 2^-ΔCT^.

### Serial dilution of DNA

Frozen-, FFPE- and Trizol-DNA were serially diluted with distilled water, TE solution (10 mM Tris-HCl/1 mM EDTA-3 Na, pH 6.0) or various concentrations of an NaCl solution (10 mM, 1 mM, 0.1 mM, or 0.01 mM). Frozen-R2 DNA was serially diluted with TE buffer or distilled water, and 0.1 volumes of 100 mM Tris-HCl/10 mM EDTA were added to the latter diluent. The dilutions were divided and measured via NanoDrop, Qubit and qPCR on the same day.

### PCR of various sizes of target DNA

PCR was performed in five regions (317, 499, 741, 1357 and 2995 bp) of the human *Golgi membrane protein 1* in 20 μl mixtures using TaKaRa Ex Taq Hot Start Version (Takara Bio). The primer sequences used are shown in S2 Table. The PCR program included initial denaturation at 94°C for 1 minute, followed by 35 cycles of 98°C for 10 seconds and 65°C for 1 minute and final extension at 72°C for 5 minutes. The PCR products (10 μl) were subjected to electrophoresis in a 2% agarose gel. For 2995-bp amplification, the combined annealing/extension step was performed at 60°C for 2 minutes, and the PCR product was electrophoresed in a 0.8% agarose gel.

### Agilent 2200 TapeStation

Frozen-DNA (H1 to H3), FFPE-DNA (H1 to H3) and Trizol-DNA (h1, h2, h4, h6 and h7) were analyzed using an Agilent 2200 TapeStation with Genomic DNA ScreenTape (Agilent Technologies, Santa Clara, CA, USA). Original DNA solutions were diluted with TE buffer to 100 ng/μl, as measured by NanoDrop. Diluted DNA samples were measured using the 2200 TapeStation according to the manufacturer’s protocol.

### Quantification of single-stranded DNA

The fluorescent dye of the Qubit ssDNA Assay Kit binds not only to ssDNA but also to dsDNA; thus, the dye by itself cannot be used to quantify ssDNA in a mixed sample of dsDNA and ssDNA. We used a Qubit dsDNA HS Assay Kit and Qubit ssDNA Assay Kit together to quantify the mixture of ssDNA and dsDNA. Deoxyribonucleic acid sodium salt from salmon testes (Sigma-Aldrich, St. Louis, MO, USA) was used as a standard dsDNA. The primer 5’- GAC AGC AAG GGT AGG GAT AG -3’ was used as a standard ssDNA. Each standard DNA was dissolved in TE buffer and subsequently diluted with TE buffer to yield a 20 ng/μl DNA solution, as determined by the DNA-50 mode of NanoDrop. The standard ssDNA solution was also quantified using the ssDNA-33 mode of NanoDrop to determine the absolute concentration of ssDNA. To prepare the standard curves for ssDNA quantification in the presence of dsDNA, various mixtures of the two DNAs (20 ng/μl each) at varying ratios were prepared (S1 Fig). The mixtures and sample DNA (20 ng/μl each) were quantified with a Qubit 3.0 fluorometer using a Qubit dsDNA HS Assay Kit and Qubit ssDNA Assay Kit according to the manufacturer’s protocols.

### Statistical analysis

Statistical analysis was performed with the paired *t*-test or Student's *t*-test. The paired t-test was applied to compare the NanoDrop with the Qubit or qPCR values, and Student's *t*-test was used to compare the ratios. A p value of <0.05 was regarded as significant.

## Results and Discussion

### Quantification of Frozen-DNA by NanoDrop, Qubit and qPCR

**Fig 1:**
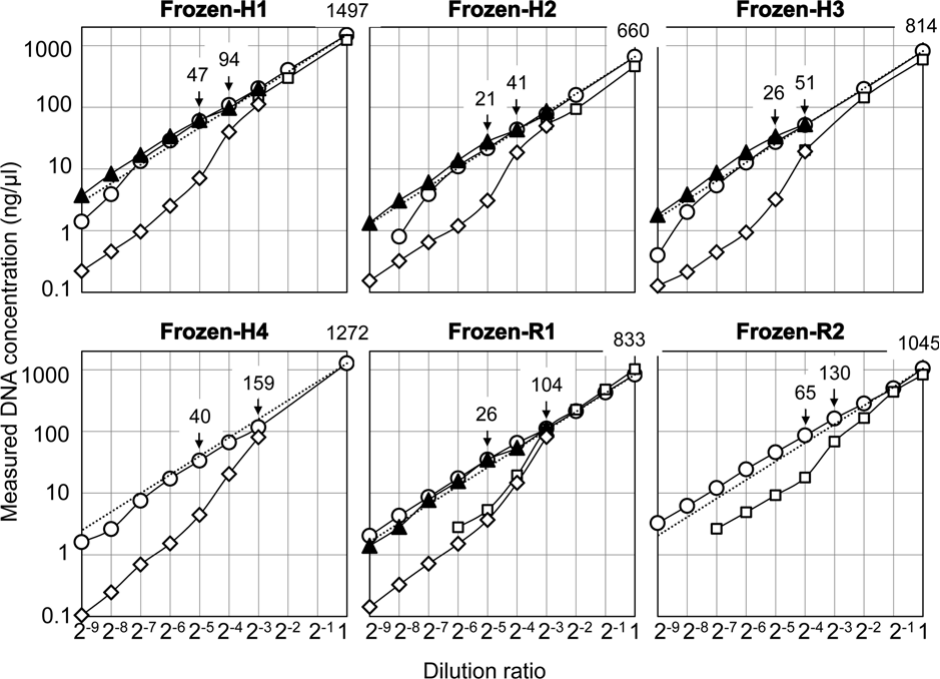
Dilution curves of Frozen-DNA diluted with distilled water as determined by NanoDrop, Qubit and qPCR. Each Frozen-DNA sample was serially diluted with distilled water, and the concentration of each diluent was measured by NanoDrop (circles), BR-Qubit (squares), HS-Qubit (diamonds) and qPCR (triangles). The broken line shows the expected NanoDrop value. The concentration (ng/μl) of each original DNA solution measured by NanoDrop is shown at the top right: dilution ratio = 1. Two additional concentrations are also shown in each graph. The detection limits of NanoDrop, BR-Qubit, HS-Qubit and qPCR are 2 ng/μl, 2 ng/μl, 0.2 ng/μl and 1 pg/μl, respectively.

**Fig 2:**
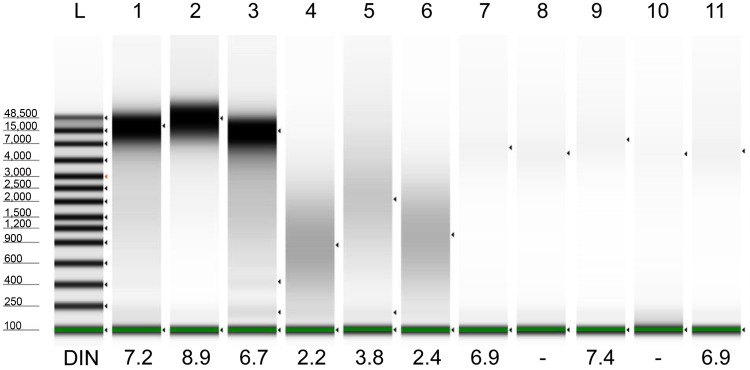
Electrophoresis of DNAs by Agilent 2200 TapeStation. DNAs (100 ng/lane) were electrophoresed on an Agilent 2200 TapeStation. The DNA integrity number (DIN) indicates the fragmentation of genomic DNA on a scale from 1 to 10. A high DIN indicates highly intact DNA, and a low DIN indicates strongly degraded DNA. Lane 1, Frozen-H1; lane 2, Frozen-H2; lane 3, Frozen-H3; lane 4, FFPE-H1; lane 5, FFPE-H2; lane 6, FFPE-H3; lane 7, Trizol-h1; lane 8, Trizol-h2; lane 9, Trizol-h4; lane 10, Trizol-h6; and lane 11, Trizol-h7.

Frozen-H1 to H4, R1 and R2 were serially diluted with distilled water, and each sample was quantified with NanoDrop and Qubit (Fig 1). We confirmed that the purities of the DNA extracts were all OD_260_/OD_280_≥1.86 and OD_260_/OD_230_≥1.97 (S1 Table) and that these Frozen-DNAs had high molecular weights (15 to 45 kbp) using an Agilent 2200 TapeStation (Fig 2). The expected NanoDrop (ex-ND) value was a theoretical value determined by multiplying the highest DNA concentration measured by NanoDrop by the dilution ratio. The values measured by NanoDrop were consistent with the ex-ND values. In contrast, those measured using Qubit were not proportional to the dilution ratio and diverged from the ex-ND values; the Qubit values were consistent with the NanoDrop values in the high concentration range but differed in the range of 1/8 to 1/32 (160 to 20 ng/μl). The dilution curve yielded by Qubit below 1/32 had the same slope as the curves yielded by the ex-ND values. Using dilutions of 11 Frozen-DNAs, we confirmed that the Qubit values were significantly lower than the NanoDrop values at an ex-ND of approximately 20 ng/μl (*p* = 2.1 x 10^−7^ by the paired *t*-test) Because a decrease in the quantity measured by fluorescence spectroscopy indicates a deterioration in DNA quality [8–11], we measured the quantity of the same serial dilution of DNA by qPCR at the same time. The qPCR quantities were similar to the ex-ND values (Frozen-H1, H2, H3 and R1 in Fig 1). These results suggest that Qubit does not always provide accurate measurements of DNA at lower concentrations, even in the absence of deterioration of DNA. The following question therefore arises: does this non-proportional decrease in Qubit values occur because of DNA dilution? As shown in Fig 1, a certain dose-dependent curve of Qubit showed three characteristic phases in the high, middle and low concentration ranges. The high and low phases exhibited the expected dose dependency. However, in the middle phase, the Qubit quantity exhibited a stronger decrease than was expected. These results suggest that another factor exists in the DNA solution and that a DNA structural transition occurs at a critical concentration of the factor. The factor is presumed to contribute to the stabilization of dsDNA; concentrations below a specific concentration of the factor induce a defined structural change in dsDNA, resulting in a second stable structure of dsDNA with a low affinity for fluorescent dye.

### Effect of salt on the quantification of Frozen-DNA by Qubit

**Fig 3:**
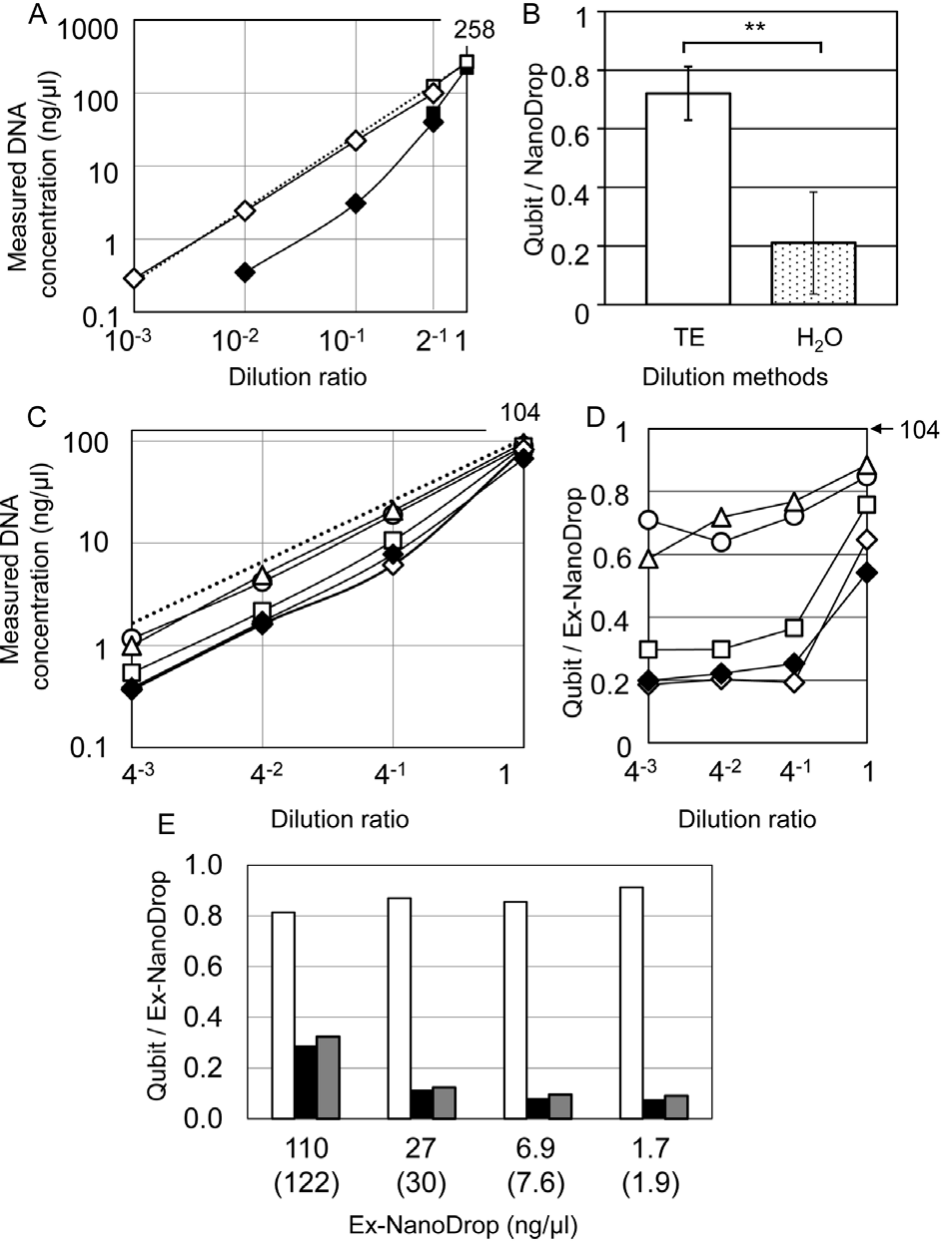
Quantification of Frozen-DNA diluted with various solutions by Qubit. (A) Frozen-R2 DNA was serially diluted with distilled water (black) or TE buffer (white), and the concentration of each diluent was measured by BR-Qubit (square) and HS-Qubit (diamond). (B) Eleven Frozen-DNAs were diluted with distilled water or TE buffer to approximately 20 ng/μl, as measured by NanoDrop, and the concentration of each diluent was measured by HS-Qubit. The ratios of the Qubit to NanoDrop values were determined for each diluent. (C, D) Frozen-R1 DNA was serially diluted with distilled water (closed diamonds), 0.01 mM NaCl (open diamonds), 0.1 mM NaCl (squares), 1 mM NaCl (triangles) or 10 mM NaCl (circles). The broken line shows the expected NanoDrop values. The concentration (ng/μl) of each original DNA solution, as measured by NanoDrop, is shown at the top right: dilution ratio = 1. (D) The Q/E ratio was determined for each diluent, as shown in Fig 3C. (E) Frozen-R2 DNA was serially diluted with TE buffer (white) or distilled water (black), and a 0.1 volume of 100 mM Tris-HCl/10 mM EDTA was added to the latter diluent (gray). The expected NanoDrop values in parentheses indicate those diluted with distilled water. The detection limits of each measurement are described in Fig 1.

To examine the effect of distilled water on the stability of DNA, Frozen-R2 DNA was serially diluted with TE buffer or distilled water, and the Qubit values of the two were then compared (Fig 3A). When DNA was diluted with TE buffer, the Qubit values were consistent with the ex-ND values and did not diverge, in contrast with DNA diluted with distilled water. We confirmed the effect of TE buffer using 11 Frozen-DNAs (Fig 3B); when original DNA solutions were diluted to approximately 20 ng/μl, as measured by NanoDrop, the ratio of the Qubit to NanoDrop values determined using TE buffer was 0.72±0.09, and that using distilled water was significantly lower. (0.21±0.17, ** *p* = 3.7 x 10^−8^ by Student's t-test) Subsequently, we investigated the effect of salt on Qubit quantification instead of TE buffer which contains Na^+^ equivalent to 3 mM (Fig 3C and 3D). When the Frozen-R1 DNA was serially diluted with 0.01 mM NaCl, the Qubit quantity diverged from the ex-ND value, which was also observed in the dilution with distilled water (Fig 3C). The ratio of the Qubit quantity to the ex-ND value (Q/E ratio) for DNA diluted with 0.01 mM NaCl was equal to that for DNA diluted in distilled water (Fig 3D). However, the Qubit quantities of DNA diluted with 1 mM NaCl and 10 mM NaCl were similar to the ex-ND values, as shown in TE buffer in Fig 3A. These results demonstrate that DNA is accurately quantified with Qubit when DNA is dissolved in 1 mM or more of NaCl and that the divergence of the Qubit quantity depends on the salt concentration; a salt concentration of 1 mM or above stabilizes dsDNA, and the Q/E ratio is consistently the highest. Below 1 mM and down to 0.01 mM, NaCl transiently destabilizes dsDNA, leading to the second conformational state of dsDNA, and the Q/E ratio then decreases and diverges from the estimated ratio. Concentrations below 0.01 mM NaCl stabilize the second conformational state of dsDNA, leading to the lowest Q/E ratio (Fig 3D).

To examine whether the structural change in DNA in the presence of a low salt concentration is reversible, salt was added to the DNA solution diluted with distilled water, and the Qubit quantity was measured before and after salt addition. The Qubit quantity was also measured for the DNA solution diluted with TE buffer, and the three Qubit values were compared with the ex-ND values (Fig 3E). The Qubit/ex-ND ratio for TE buffer was consistently greater than 0.8. In contrast, DNA was not recovered at low ratios of 0.1 to 0.3 regardless of whether salt was added. These results suggest that the dilution of DNA in the absence of NaCl causes irreversible structural changes to the DNA. Thus, to accurately evaluate Frozen-DNA, the Qubit measurement methods require the preparation of DNA samples in the presence of at least 1 mM NaCl at the dilution stage.

What is the structural difference between the two states of dsDNA at salt concentrations of greater than 1 mM and less than 0.1 mM? Korolev N et al. demonstrated that cations reduced the electrostatic repulsion of negatively charged DNA phosphates and condensed DNA. The electrostatic free energy was proportional to the Na^+^ concentration [15]. Although the binding mechanism of fluorescent dye of Qubit to dsDNA is unknown, it is possible that a certain extension of the dsDNA structure occurs under the low-salt conditions to hinder binding with fluorescent dye. Widom J et al. have indicated that the extension of dsDNA is very rapidly induced by dilution of cations, whereas the kinetics of condensation are slow, ranging from minutes to hours [16, 17]. The slow velocity of dsDNA condensation by cations may be a cause of the apparent irreversible change in the affinity of dsDNA for fluorescent dye in our study.

### Quantification of FFPE-DNA by NanoDrop, Qubit and qPCR

**Fig 4:**
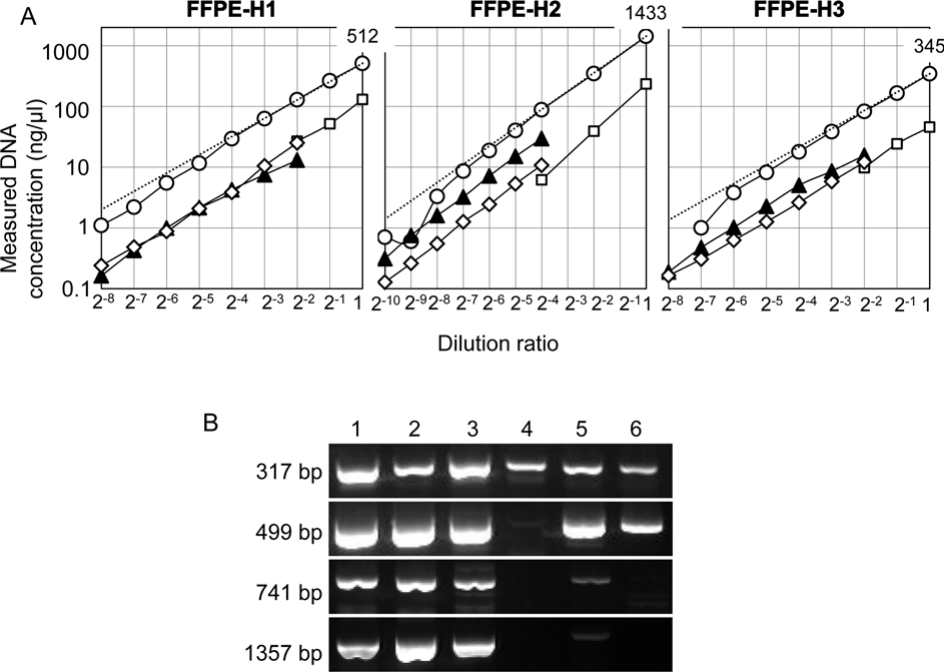
Quantification and qualification of FFPE-DNA. (A) Each FFPE-DNA was serially diluted with distilled water, and the concentration of each diluent was measured by NanoDrop (circles), BR-Qubit (squares), HS-Qubit (diamonds) and qPCR (triangles). The broken line indicates the expected NanoDrop value. The concentration (ng/μl) of each original DNA solution, as measured by NanoDrop, is shown at the top right: dilution ratio = 1. The detection limits of each measurement are described in Fig 1. (B) Various lengths of the target sequence were amplified from Frozen- and FFPE-DNAs. The amplified products were electrophoresed on agarose gels. Lane 1, Frozen-H1; lane 2, Frozen-H2; lane 3, Frozen-H3; lane 4, FFPE-H1; lane 5, FFPE-H2; and lane 6, FFPE-H3.

Similar to Frozen-DNA, FFPE-H1–H3 was serially diluted with distilled water, and each sample was quantified with NanoDrop, Qubit and qPCR (Fig 4A). The Qubit and qPCR values were proportional to the dilution ratios and were significantly lower than the ex-ND values (*p* = 0.01 and 0.003, respectively), even at the highest concentration. These results indicate that the divergence of the Qubit quantity of FFPE-DNA is likely due to a deterioration in DNA quality such as the degradation and modification characteristics of FFPE-DNA itself. In this respect, Qubit is superior to NanoDrop for quantifying FFPE-DNA.

Three measurements of FFPE-DNA indicated another issue. The ratios of Qubit/ex-ND were similar among FFPE-DNAs at 0.12±0.02. In contrast, the ratio of the qPCR quantity to the ex-ND value (qPCR/ex-ND) was different for each FFPE-DNA as follows: 0.1 in FFPE-H1, 0.3 in FFPE-H2 and 0.2 in FFPE-H3. When various lengths of DNA were amplified from the three FFPE-DNAs, the amplification efficiency varied significantly (Fig 4B). FFPE-H1, FFPE-H2, and FFPE-H3 yielded amplification products of no longer than 499 bp, 1357 bp, and 741 bp. Thus, the most fragmented DNA was FFPE-H1, followed in order by FFPE-H3 and FFPE-H2, which was consistent with the qPCR quantities.

The size distribution of 100 ng of DNA was also analyzed by electrophoresis using an Agilent 2200 TapeStation (Fig 2 and S2 Fig). FFPE-DNAs showed a smear, and their DNA integrity numbers (DINs) were 2.2, 3.8 and 2.4 for FFPE-H1, FFPE-H2 and FFPE-H3, respectively, consistent with the qPCR quantities. These results indicate that qPCR quantifies the fragmentation of DNA, whereas Qubit does not; indeed, Qubit does not always accurately evaluate PCR-amplifiable DNA. Therefore, the divergence of the Qubit quantity of FFPE-DNA from the ex-ND value is not due to degradation but likely to a modification, such as an irreversible conformational change of dsDNA caused by formalin fixation. Thus, the qPCR method is the most suitable method for estimation of the degree of fragmentation.

### Quantification of Trizol-DNA by NanoDrop, Qubit and qPCR

Human biopsy materials are very rare and provide few samples for study. The simultaneous extraction of DNA, RNA and proteins from a single sample would be useful for elucidating mechanisms underlying the interaction and control of gene expression. DNA and proteins are extracted from the fractions remaining after RNA extraction. In the present study, the DNA extracted from this fraction (Trizol-DNA) was compared with Frozen-DNA and FFPE-DNA.

**Fig 5:**
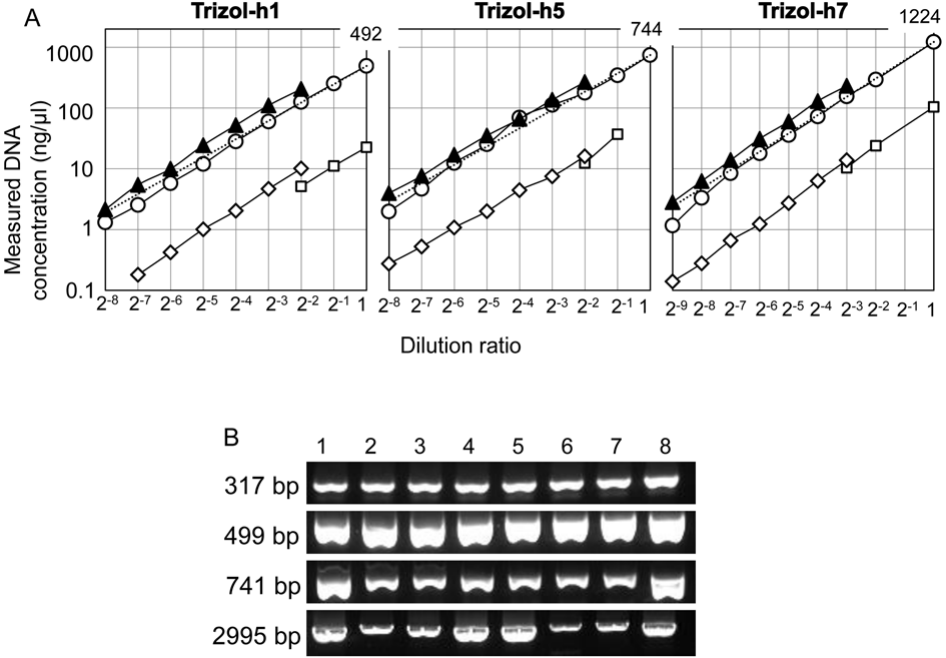
Quantification and qualification of Trizol-DNA. (A) Trizol-DNA was serially diluted with distilled water, and the concentration of each diluent was measured by NanoDrop (circles), BR-Qubit (squares), HS-Qubit (diamonds) and qPCR (triangles). The broken line indicates the expected NanoDrop value. The concentration (ng/μl) of each original DNA solution, as measured by NanoDrop, is shown at the top right: dilution ratio = 1. The detection limits of each measurement are described in Fig 1. (B) Various lengths of the target sequence were amplified from Trizol-DNAs, and the amplified products were electrophoresed on an agarose gel. Lane 1, Frozen-H1; lane 2, Trizol-h1; lane 3, Trizol-h2; lane 4, Trizol-h3; lane 5, Trizol-h4; lane 6, Trizol-h5; lane 7, Trizol-h6; and lane 8, Trizol-h7.

Similar to Frozen-DNA and FFPE-DNA, Trizol-h1, h5 and h7 were serially diluted with distilled water, and each sample was quantified with NanoDrop, Qubit and qPCR (Fig 5A). The NanoDrop quantity was similar to the ex-ND value, whereas the Qubit value was significantly lower than the ex-ND value (*p* = 0.01), as observed for FFPE-DNA. These results indicate that the divergence of the Qubit quantity of Trizol-DNA likely depends on the nature of Trizol-DNA itself. The qPCR quantity was higher than the ex-ND value. The qPCR/ex-ND ratios were 1.51 in Trizol-h1, 1.43 in Trizol-h5 and 1.48 in Trizol-h7. The qPCR/ex-ND ratio of Trizol-DNAs was significantly higher than that of Frozen-DNAs (*p* = 0.047). Thus, DNA extracted with Trizol easily served as a template for amplification. When various lengths of DNA, ranging from 317 bp to 2995 bp, were amplified from the three Trizol-DNAs, all of the Trizol-DNAs as well as the Frozen-DNAs exhibited similar frequencies of amplification (Fig 5B). Therefore, Trizol-DNA was not fragmented to less than 2995 bp.

However, the electrophoresis results using an Agilent 2200 TapeStation showed that Trizol-DNAs were weakly stained, appearing as only slightly visible smears (Fig 2 and S2 Fig). Because SYBR Green I was used for staining DNA for the 2200 TapeStation, these results indicate that the dsDNA concentrations were markedly reduced in Trizol-DNAs. We also confirmed the results by performing agarose gel electrophoresis and statistical analysis of the fluorescence densitometry data (S3 Fig). As shown in Fig 2, the DINs of Trizol-DNAs were high, although the remaining dsDNAs were outside of the quantitation range. Figs 2 and 5 show that almost all of the Trizol-DNA was denatured into ssDNA but not fragmented. Kotorashvili A et al. have also observed a similar smear pattern of Trizol-DNA in agarose gel electrophoresis [18].

**Fig 6:**
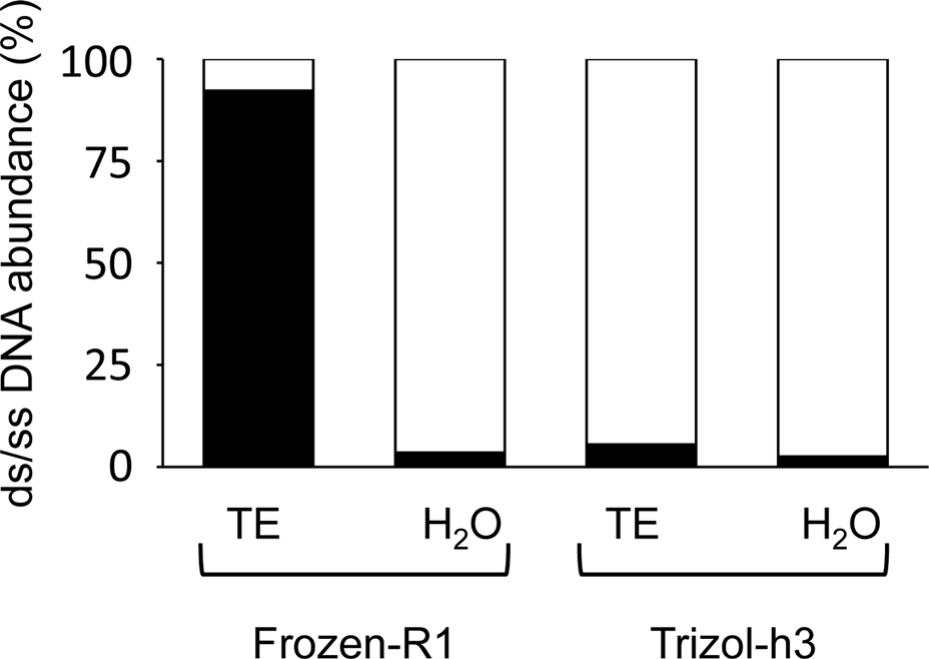
Quantification of ssDNA by ssDNA-Qubit and dsDNA-Qubit. Frozen-R1 and Trizol-h3 diluted with TE buffer or distilled water in 20 ng/μl were measured with ssDNA-Qubit and dsDNA-Qubit. The amounts of dsDNA (black) and ssDNA (white) were determined using the standard curve shown in S1 Fig.

Next, we attempted to quantify ssDNA. The mixtures containing various ratios of ssDNA to dsDNA were quantified with ssDNA-Qubit and dsDNA-Qubit, and standard curves were prepared (S1 Fig). Then, we determined the ssDNA concentrations using the standard curves. When Frozen-R1 was diluted with TE buffer, dsDNA accounted for 92.3% of the total DNA. When it was diluted with distilled water, the ssDNA concentration was 96.5% (Fig 6). Thus, Frozen-DNA was denatured into ssDNA during the dilution stage with distilled water. However, ssDNA in Trizol-h3 accounted for 94.5–97.5% of the sample regardless of the dilution. This result indicates that Trizol-DNA was already denatured into ssDNA after the extraction step.

Majumdar G et al. also characterized Trizol-DNA, which was dissolved with NaOH-Hepes buffer according to the manufacturer’s protocol. They concluded that contaminated DNA was recovered and that the yield of genomic DNA was very poor because of a very weak DNA signal detected on agarose gel electrophoresis, which was also observed in the present study [19]. Although we dissolved DNA with distilled water instead of NaOH-Hepes buffer, both Trizol-DNAs were likely to form ssDNA and yielded similar gel images in electrophoresis. Therefore, the denaturation of Trizol-DNA may be caused by a main component of Trizol reagent, such as guanidine salt, which disrupts the hydrogen bonding network and denatures RNase.

## Conclusion

DNA quantification by Qubit is occasionally underestimated depending on the method used for DNA extraction and dilution (Table 1). To accurately quantify DNA, we should consider the following three key points: 1) Qubit quantification of Frozen-DNA requires a sufficient concentration of salt in the DNA solution; 2) qPCR quantification of FFPE-DNA is more accurate than Qubit quantification; and 3) Qubit quantification of Trizol-DNA is apparently underestimated because Trizol-DNA is mostly denatured.

**Table 1:** Recommended quantification methods for DNA available for PCR.

	Quantification		
DNA	NanoDrop	Qubit	qPCR
**Frozen**	suitable	suitable*	suitable
**FFPE**	overestimated	suitable	most suitable
**Trizol**	suitable	underestimated	suitable

* Depending on salt concentration.

## Supporting Information

S1 Fig Standard curves of single-stranded DNA quantification. The table shows how to prepare various mixtures of dsDNA and ssDNA using 20 ng/μl of each, as determined by the dsDNA mode (DNA-50) of NanoDrop. The absolute concentration of ssDNA was determined by the ssDNA mode (ssDNA-33), and that of each standard solution is shown in parentheses. After measuring the mixtures with Qubit dsDNA (diamonds) and Qubit ssDNA (circles), standard curves were prepared (lower panel).
(PDF)

S2 Fig Fluorescence electropherogram of Frozen-DNA, FFPE-DNA and Trizol-DNA measured with a 2200 TapeStation. The gel image of Fig 2 is presented as an electropherogram overlay.
(PDF)

S3 Fig Agarose gel electrophoresis of Frozen-DNA, FFPE-DNA and Trizol-DNA with SYBR Green I staining. (A) DNAs (500 ng/lane) were electrophoresed on an agarose gel and stained with SYBR Green I. Lane 1, Frozen-H1; lane 2, Frozen-H2; lane 3, Frozen-H3; lane 4, FFPE-H1; lane 5, FFPE-H2; lane 6, FFPE-H3; lane 7, Trizol-h1; lane 8, Trizol-h6; and lane 9, Trizol-h7. (B) The fluorescence intensity of each lane was measured using a CS Analyzer 3 (Atto Co., Tokyo, Japan) and is expressed as a value relative to Frozen-H1 intensity. The mean and standard deviation of relative fluorescence were determined for each group of Frozen-DNAs (lanes 1 to 3), FFPE-DNAs (lanes 4 to 6) and Trizol-DNAs (lanes 7 to 9). The fluorescence intensities of dsDNA in Trizol-DNAs were significantly lower than those of Frozen-DNAs (*p = 0.025) and FFPE-DNAs (**p = 0.002, by Student’s *t*-test).
(PDF)

S1 Table Primer sequences used in this study. (PDF)

S2 Table NanoDrop data of original DNA solutions. (PDF)

## References

[1] Bianchi DW. From prenatal genomic diagnosis to fetal personalized medicine: progress and challenges. Nature medicine. 2012;18(7):1041–51. 10.1038/nm.2829 22772565 PMC4433004

[2] Hahn S, Lapaire O, Tercanli S, Kolla V, Hosli I. Determination of fetal chromosome aberrations from fetal DNA in maternal blood: has the challenge finally been met? Expert reviews in molecular medicine. 2011;13:e16 10.1017/S1462399411001852 21542948 PMC3087311

[3] Chiu RW, Cantor CR, Lo YM. Non-invasive prenatal diagnosis by single molecule counting technologies. Trends in genetics: TIG. 2009;25(7):324–31. 10.1016/j.tig.2009.05.004 . 19540612

[4] Forshew T, Murtaza M, Parkinson C, Gale D, Tsui DW, Kaper F, et al Noninvasive identification and monitoring of cancer mutations by targeted deep sequencing of plasma DNA. Science translational medicine. 2012;4(136):136ra68 10.1126/scitranslmed.3003726 . 22649089

[5] Murtaza M, Dawson SJ, Tsui DW, Gale D, Forshew T, Piskorz AM, et al Non-invasive analysis of acquired resistance to cancer therapy by sequencing of plasma DNA. Nature. 2013;497(7447):108–12. 10.1038/nature12065 . 23563269

[6] Singer VL, Jones LJ, Yue ST, Haugland RP. Characterization of PicoGreen reagent and development of a fluorescence-based solution assay for double-stranded DNA quantitation. Anal Biochem. 1997;249(2):228–38. 10.1006/abio.1997.2177 . 9212875

[7] Green MR, Sambrook J. Molecular cloning: a laboratory manual (fourth edition). Cold Spring Harbor, N.Y.: Cold Spring Harbor Laboratory Press; 2012 pp. 6–9.

[8] Georgiou CD, Papapostolou I. Assay for the quantification of intact/fragmented genomic DNA. Anal Biochem. 2006;358(2):247–56. 10.1016/j.ab.2006.07.035 . 16942746

[9] Sedlackova T, Repiska G, Celec P, Szemes T, Minarik G. Fragmentation of DNA affects the accuracy of the DNA quantitation by the commonly used methods. Biol Proced Online. 2013;15(1):5 10.1186/1480-9222-15-5 23406353 PMC3576356

[10] Shokere LA, Holden MJ, Jenkins GR. Comparison of fluorometric and spectrophotometric DNA quantification for real-time quantitative PCR of degraded DNA. Food Control. 2009;20(4):391–401. 10.1016/j.foodcont.2008.07.009 .

[11] Holden MJ, Haynes RJ, Rabb SA, Satija N, Yang K, Blasic JR Jr. Factors affecting quantification of total DNA by UV spectroscopy and PicoGreen fluorescence. J Agric Food Chem. 2009;57(16):7221–6. 10.1021/jf901165h . 19627145

[12] Klein D. Quantification using real-time PCR technology: applications and limitations. Trends in Molecular Medicine. 2002;8(6):257–60. Pii S1471-4914(02)02355-9 10.1016/S1471-4914(02)02355-9 . 12067606

[13] Simbolo M, Gottardi M, Corbo V, Fassan M, Mafficini A, Malpeli G, et al DNA qualification workflow for next generation sequencing of histopathological samples. PLoS One. 2013;8(6):e62692 10.1371/journal.pone.0062692 23762227 PMC3675123

[14] Green MR, Sambrook J. Molecular cloning: a laboratory manual (fourth edition). Cold Spring Harbor, N.Y.: Cold Spring Harbor Laboratory Press; 2012 pp. 47–53.

[15] Korolev N, Lyubartsev AP, Nordenskiold L. Application of polyelectrolyte theories for analysis of DNA melting in the presence of Na+ and Mg2+ ions. Biophysical journal. 1998;75(6):3041–56. 10.1016/S0006-3495(98)77745-8 9826624 PMC1299975

[16] Widom J, Baldwin RL. Cation-Induced Toroidal Condensation of DNA Studies with Co3+(Nh3)6. J Mol Biol. 1980;144(4):431–53. 10.1016/0022-2836(80)90330-7 . 6454789

[17] Bloomfield VA. DNA condensation by multivalent cations. Biopolymers. 1997;44(3):269–82. 10.1002/(SICI)1097-0282(1997)44:3<269::AID-BIP6>3.0.CO;2-T . 9591479

[18] Kotorashvili A, Ramnauth A, Liu C, Lin J, Ye K, Kim R, et al Effective DNA/RNA co-extraction for analysis of microRNAs, mRNAs, and genomic DNA from formalin-fixed paraffin-embedded specimens. PLoS One. 2012;7(4):e34683 10.1371/journal.pone.0034683 22514653 PMC3326040

[19] Majumdar G, Vera S, Elam MB, Raghow R. A streamlined protocol for extracting RNA and genomic DNA from archived human blood and muscle. Anal Biochem. 2015;474:25–7. 10.1016/j.ab.2014.12.021 . 25579785

