# Supplementary material for: Pitfalls of DNA Quantification Using DNA-Binding Fluorescent Dyes and Suggested Solutions
Source: PLoS One. 2016 Mar 3;11(3):e0150528. doi: 10.1371/journal.pone.0150528 (PMC4777359; doi:10.1371/journal.pone.0150528)
Supplement: S1 Fig — The table shows how to prepare various mixtures of dsDNA and ssDNA using 20 ng/μl of each, as determined by the dsDNA mode (DNA-50) of NanoDrop. The absolute concentration of ssDNA was determined by the ssDNA mode (ssDNA-33), and that of each standard solution is shown in parentheses. After measuring the mixtures with Qubit dsDNA (diamonds) and Qubit ssDNA (circles), standard curves were prepared (lower panel). (PDF) [file pone.0150528.s001.pdf]

Standard	NanoDrop	Mixture										
		1	2	3	4	5	6	7	8	9	10	11
dsDNA (ng/µl)	DNA-50	20.2	17.3	14.7	12.3	10.1	8.1	6.3	4.5	2.9	1.4	0.0
ssDNA (ng/µl)	DNA-50	0.0	2.8	5.4	7.8	10.0	12.0	13.9	15.6	17.2	18.7	20.1
	ssDNA-33	(0.0)	(1.9)	(3.7)	(5.3)	(6.8)	(8.1)	(9.4)	(10.5)	(11.6)	(12.7)	(13.6)

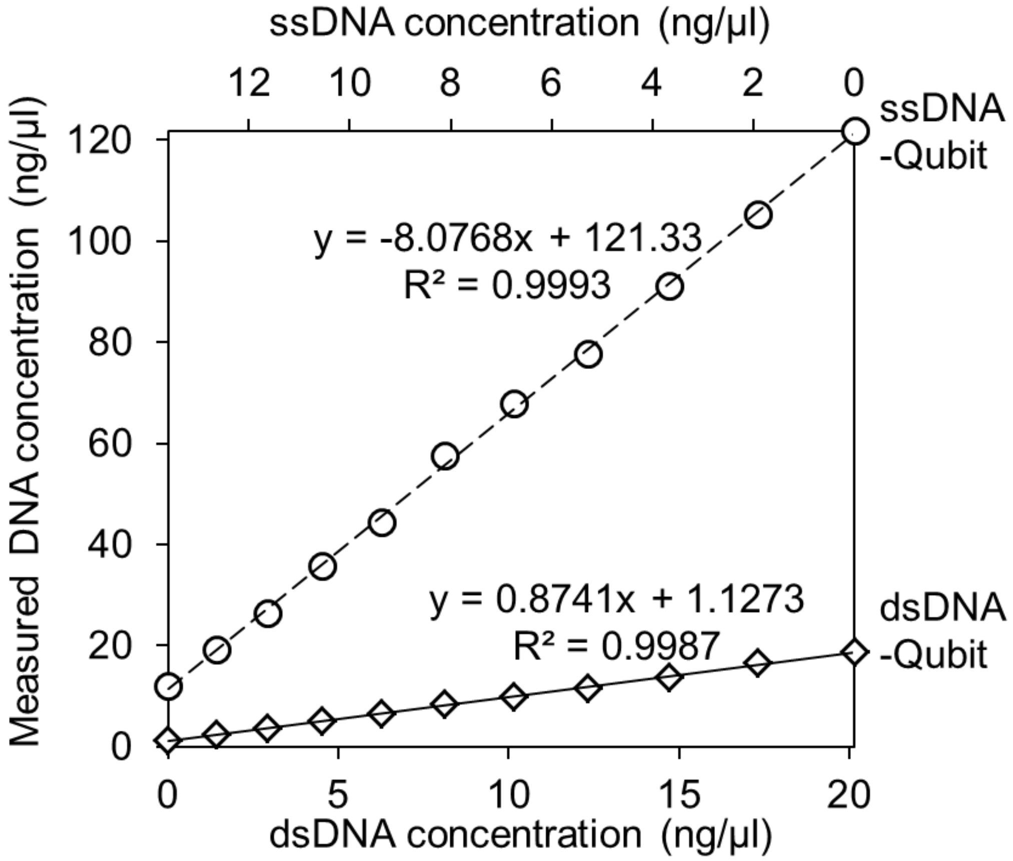

S1 Fig. Standard curves of single-stranded DNA quantification

The table shows how to prepare various mixtures of dsDNA and ssDNA using 20 ng/µl of each, as determined by the dsDNA mode (DNA-50) of NanoDrop. The absolute concentration of ssDNA was determined by the ssDNA mode

(ssDNA-33), and that of each standard solution is shown in parentheses. After measuring the mixtures with Qubit dsDNA (diamonds) and Qubit ssDNA (circles), standard curves were prepared (lower panel).
