# Supplementary material for: Pitfalls of DNA Quantification Using DNA-Binding Fluorescent Dyes and Suggested Solutions
Source: PLoS One. 2016 Mar 3;11(3):e0150528. doi: 10.1371/journal.pone.0150528 (PMC4777359; doi:10.1371/journal.pone.0150528)
Supplement: S2 Fig — The gel image of Fig 2 is presented as an electropherogram overlay. (PDF) [file pone.0150528.s002.pdf]

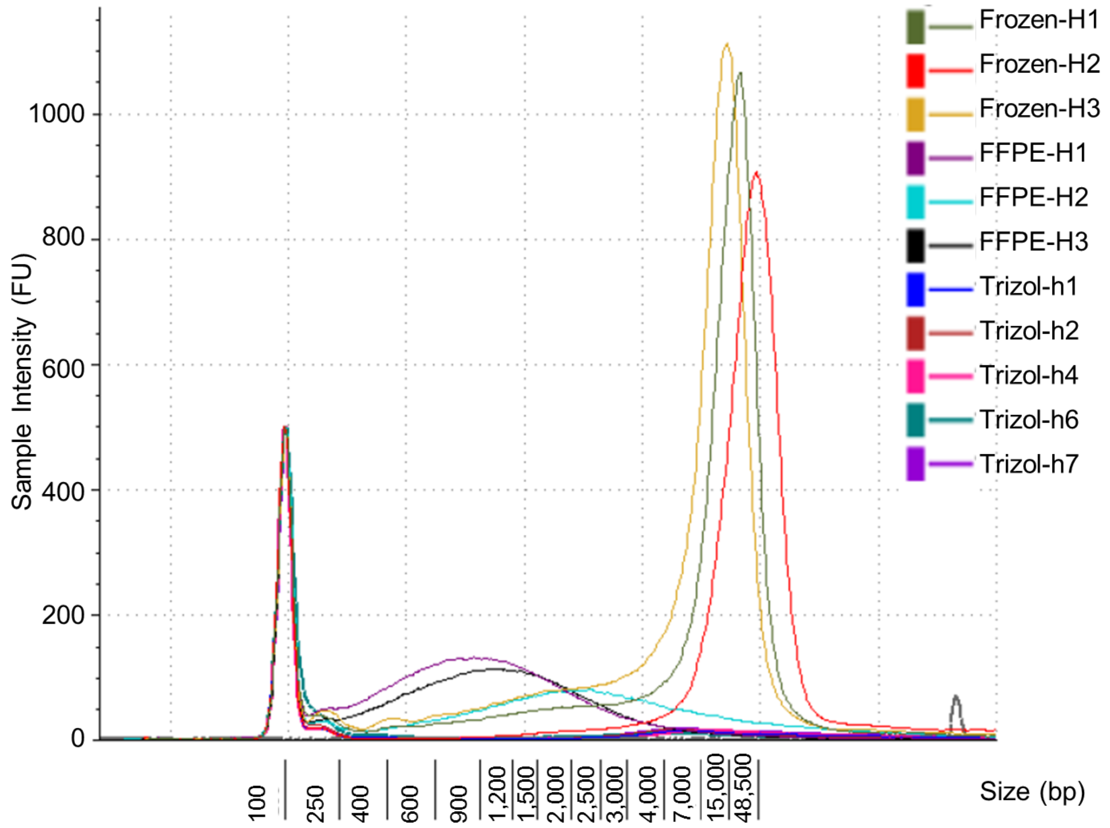

S2 Fig. Fluorescent electropherogram of Frozen-DNA, FFPE-DNA and Trizol-DNA measured by the 2200 TapeStation.

The gel image of Fig 2 is presented as an electropherogram overlay.
