# Supplementary material for: Pitfalls of DNA Quantification Using DNA-Binding Fluorescent Dyes and Suggested Solutions
Source: PLoS One. 2016 Mar 3;11(3):e0150528. doi: 10.1371/journal.pone.0150528 (PMC4777359; doi:10.1371/journal.pone.0150528)
Supplement: S3 Fig — (A) DNAs (500 ng/lane) were electrophoresed on an agarose gel and stained with SYBR Green I. Lane 1, Frozen-H1; lane 2, Frozen-H2; lane 3, Frozen-H3; lane 4, FFPE-H1; lane 5, FFPE-H2; lane 6, FFPE-H3; lane 7, Trizol-h1; lane 8, Trizol-h6; and lane 9, Trizol-h7. (B) The fluorescence intensity of each lane was measured using a CS Analyzer 3 (Atto Co., Tokyo, Japan) and is expressed as a value relative to Frozen-H1 intensity. The mean and standard deviation of relative fluorescence were determined for each group of Frozen-DNAs (lanes 1 to 3), FFPE-DNAs (lanes 4 to 6) and Trizol-DNAs (lanes 7 to 9). The fluorescence intensities of dsDNA in Trizol-DNAs were significantly lower than those of Frozen-DNAs (*p = 0.025) and FFPE-DNAs (**p = 0.002, by Student’s t-test). (PDF) [file pone.0150528.s003.pdf]

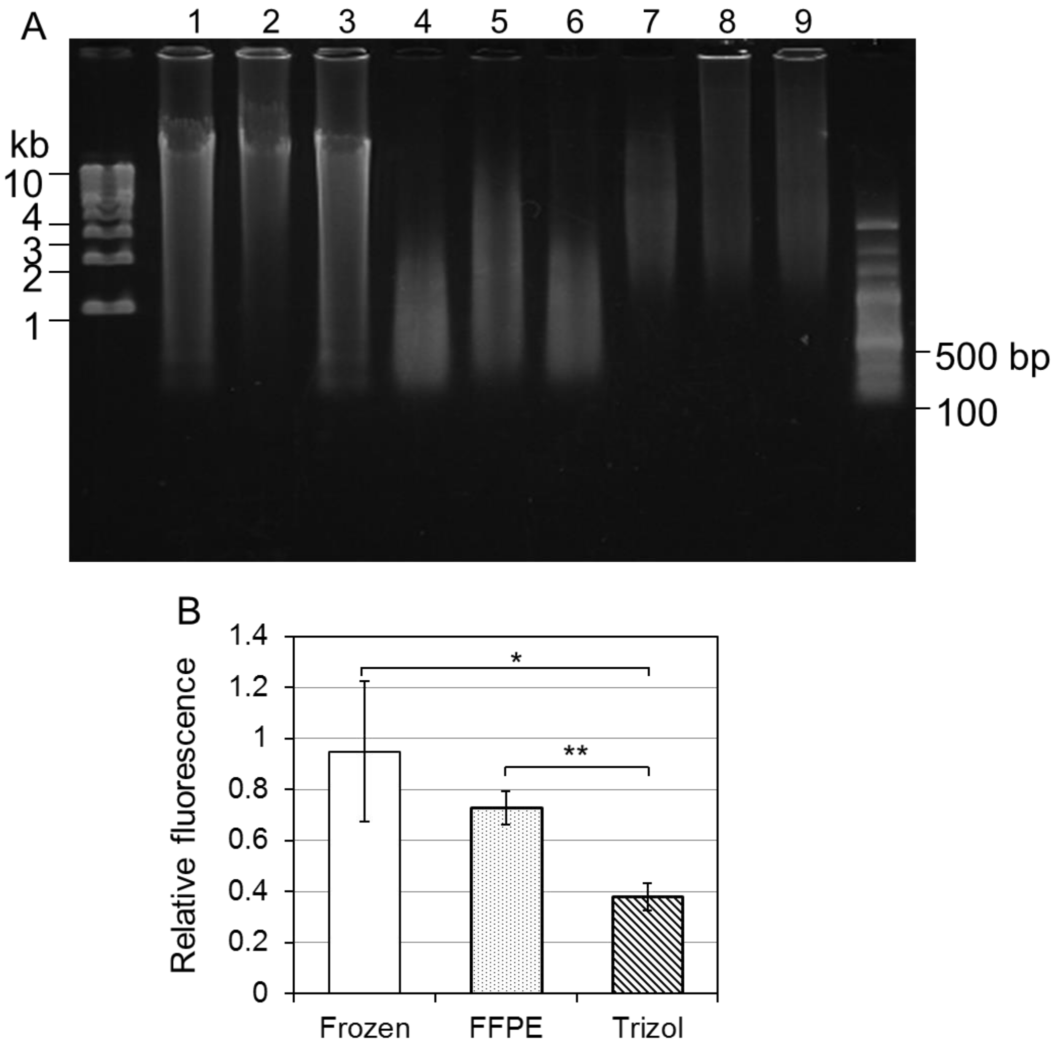

S3 Fig. Agarose gel electrophoresis of Frozen-DNA, FFPE-DNA and Trizol-DNA with SYBR Green I staining.

(A) DNAs (500 ng/lane) were electrophoresed on an agarose gel and stained with SYBR Green I. Lane 1, Frozen-H1; lane 2, Frozen-H2; lane 3, Frozen-H3; lane 4, FFPE-H1; lane 5, FFPE-H2; lane 6, FFPE-H3; lane 7, Trizol-h1; lane 8, Trizol-h6; and lane 9, Trizol-h7. (B) The fluorescence intensity of each lane was measured using a CS Analyzer 3 (Atto Co., Tokyo, Japan) and is expressed as a value relative to Frozen-H1 intensity. The mean and standard deviation of

relative fluorescence were determined for each group of Frozen-DNAs (lanes 1 to 3), FFPE-DNAs (lanes 4 to 6) and Trizol-DNAs (lanes 7 to 9). The fluorescence intensities of dsDNA in Trizol-DNAs were significantly lower than those of Frozen-DNAs (*p=0.025) and FFPE-DNAs (**p=0.002, by Student's *t*-test).
