# Supplementary material for: Pitfalls of DNA Quantification Using DNA-Binding Fluorescent Dyes and Suggested Solutions
Source: PLoS One. 2016 Mar 3;11(3):e0150528. doi: 10.1371/journal.pone.0150528 (PMC4777359; doi:10.1371/journal.pone.0150528)
Supplement: S1 Table — (PDF) [file pone.0150528.s004.pdf]

S1 Table. Primer sequences used in this study

PCR length (bp)	Primer sequence (5'-3')	
317	F	GGA TGA TAC GGT CTG CTT CG
	R	TGG CTG GCA ATA AAC AAC AA
499	F	CTA CCA GCC CAC GCT TCT T
	R	AAC CGC AAT CCT ACA CAA CC
741	F	CCC CTG GTG TAA AAG GCT CT
	R	TTC TGT AAG CAG CAA TGT TTG
1357	F	= 741F
	R	= 317R
2995	F	= 741F
	R	GTG GCT GGA CTG GAT TTT G
