# Supplementary material for: Pitfalls of DNA Quantification Using DNA-Binding Fluorescent Dyes and Suggested Solutions
Source: PLoS One. 2016 Mar 3;11(3):e0150528. doi: 10.1371/journal.pone.0150528 (PMC4777359; doi:10.1371/journal.pone.0150528)
Supplement: S2 Table — (PDF) [file pone.0150528.s005.pdf]

S2 Table. NanoDrop data of original solutions of DNA

DNA	Conc (ng/μl)	$OD_{260/280}$	$OD_{260/230}$
**Frozen-H1**	1497	1.89	2.06
**Frozen-H2**	660	1.93	2.06
**Frozen-H3**	815	1.88	1.97
**Frozen-H4**	1273	1.92	2.18
**Frozen-H5**	409	1.86	2.08
**Frozen-H6**	359	1.86	2.07
**Frozen-R1**	834	1.90	2.33
**Frozen-R2**	1046	1.90	2.35
**Frozen-R3**	1082	1.89	2.30
**Frozen-R4**	1137	1.88	2.38
**Frozen-R5**	922	1.89	2.38
**FFPE-H1**	512	1.76	1.66
**FFPE-H2**	1433	1.81	1.36
**FFPE-H3**	345	1.74	1.22
**Trizol-h1**	492	1.82	2.05
**Trizol-h2**	328	1.91	2.19
**Trizol-h3**	441	1.83	2.25
**Trizol-h4**	428	1.85	2.15
**Trizol-h5**	744	1.87	1.28
**Trizol-h6**	419	1.88	0.87
**Trizol-h7**	1224	1.90	2.07
